# Enhancing synbiotic dairy beverages with chemically cross-linked inulin for improved texture and stability

**DOI:** 10.1007/s13197-025-06243-w

**Published:** 2025-03-07

**Authors:** Hatice Oto, H. Ceren Akal, Gökçe Eminoğlu

**Affiliations:** Faculty of Agriculture Department of Dairy Technology, Ankara University, Diskapi, Ankara, 06110 Turkey

**Keywords:** Cross-linking, Inulin, Rheology, Synbiotic

## Abstract

In this study synbiotic dairy beverages containing chemically cross-linked inulin (using sodium hexametaphosphate-SHMP) and three probiotic bacteria strains (*Lactobacillus (Lb.) acidophilus*, *Lacticaseibacillus (Lcb.) rhamnosus*, and *Lcb. paraparacasei*) were examined during 30 days of storage. The samples were compared with control samples produced using natural inulin and the effects of cross-linked inulin on the composition, acidity, microbiological, physical (colour, water holding capacity, rheology), electrophoretic profile (SDS-PAGE), total phenolic content, antioxidant capacity and sensory (appearance, structure, flavour) properties of the synbiotic dairy beverages were determined. This study demonstrated that cross-linked inulin enhanced viscosity and water-holding capacity resulting in a more stable and thicker product without significantly altering the compositional properties or probiotic viability. Cross-linking inulin did not impact the viability of probiotic bacteria, which remained above 10^6^ CFU/mL. While the amount of total phenolic compounds was not affected by the different probiotics and inulin, the probiotics and cross-linked inulin did influence the antioxidant capacity of the samples. Sensory evaluation indicated that all samples were well-received and acceptable. These findings demonstrated that incorporating cross-linked inulin in synbiotic dairy beverage production enhanced the rheological properties without significantly affecting other analyzed characteristics of samples. There is no previous study on dairy beverages produced with cross-linked inulin. Therefore, this is the first study to systematically evaluate the effect of chemically cross-linked inulin on the textural and sensory properties of synbiotic beverages.

**Supplementary Information:**

The online version contains supplementary material available at 10.1007/s13197-025-06243-w.

## Introduction

Nutrition science has evolved from its initial focus on preventing deficiencies and promoting physical development to a more comprehensive understanding of diet’s impact on health and well-being. This shift has led to a focus on healthier food options, particularly functional foods. These are defined as foods that contain biologically active compounds that provide proven health benefits for the prevention, management or treatment of diseases or their symptoms (Baker et al. 2022). Synbiotic foods, which combine prebiotics and probiotics, represent one of the most widely consumed and recognized categories of functional foods. They are increasingly popular among health-conscious consumers.

Inulin, a key component in many synbiotic products, is a linear or branched fructose polymer found in the tubers and roots of over 36,000 plant species. Inulin is primarily sourced from plants such as artichoke, chicory, onion, asparagus, garlic, banana, barley, and wheat. Inulin has health benefits such as showing prebiotic properties, reducing cardiometabolic risks (Mitchell et al. 2015), improving bone metabolism (Drabinska et al. 2019), and protecting against colon cancer (Ali et al. 2019). Beyond its health benefits, inulin exhibits valuable technological properties, particularly in enhancing the physical characteristics of foods. These dual advantages – health benefits and technological effects – have led to its widespread preference in the food industry.

The effect of inulin has been investigated in different studies in order to provide synbiotic properties in the production of dairy beverages. However, while inulin successfully enhances textural properties in cheese, yogurt, and ice cream, its effect on physical properties in liquid products has been inconsistent (Fornelli et al. 2015). Some studies have reported that adding inulin to dairy-based beverages improve viscosity and physical properties of the product (da Silveira et al. 2015; Guimarães et al. 2018), while others have reported that textural properties are not affected by inulin (Fornelli et al. 2015; Montanuci et al. 2012). This variability suggests that inulin’s impact on sensory properties in liquid products may be influenced by factors such as stability and interactions with other ingredients, despite its proven benefits. Because dairy-based drinks contain components such as proteins and lipids that can interact with inulin, affecting its stability (Arruda et al. 2020). To address these challenges and fully harness advantages of inulin, cross-linking has emerged as a potential alternative method. The cross-linking process increases the resistance to enzymatic reactions. This makes the structure more stable and thus strengthens the technological effects of inulin. Additionally, cross-linking increases molecular weight of inulin, which prolongs its renal excretion time compared to natural inulin’s rapid excretion due to its lower molecular weight (Li et al. 2010).

The literature on cross-linked inulin is currently limited, but existing studies have yielded promising results. Li et al. (2019a) investigated the effects of cross-linking on inulin with varying degrees of polymerization. This research demonstrated that cross-linking positively influenced rheological properties and increased viscosity in inulin dispersions. In another study, Akal (2023) examined the effect of different cross-linking agent (SHMP) concentrations and various conditions (concentration, temperature) on inulin properties. This research corroborated the findings of Li et al. (2019a), confirming the positive impact of cross-linking on rheological properties and viscosity of inulin dispersions. Furthermore, Li et al. (2019b) reported that incorporating cross-linked inulin in yogurt production increased the firmness of the samples. These findings collectively suggest that cross-linked inulin could offer enhanced functional properties in food applications, potentially addressing some of the limitations observed with natural inulin in liquid food systems. Research has also indicated that adding cross-linked inulin positively influences the viability of yogurt bacteria. Although studies have reported that cross-linked inulin affects rheological properties, these studies have mostly been performed in model systems (as solutions in aqueous systems). The limited number of studies in the literature on the use of cross linked prebiotics in complex systems such as food and beverage makes this study unique.

The application of cross-linked inulin in beverage products, particularly synbiotic dairy beverages, is considered potentially significant. The importance of this study lies in addressing stability challenges and consistency requirements in synbiotic dairy beverages. Therefore, this study aims to assess the impact of incorporating cross-linked inulin in synbiotic dairy beverage production. The research focused on evaluating the effects on the general properties of beverages, with a particular emphasis on rheological characteristics. This investigation seeks to address the current knowledge gap regarding cross-linked inulin’s potential benefits in liquid food systems, potentially offering new insights for improving the quality and stability of synbiotic dairy beverages.

## Materials and methods

### Materials

The raw bovine milk (12.1 ± 0.2 dry matter, 3.5 ± 0.3% protein, 3.6 ± 0.1% fat, 0.61 ± 0.03% ash, 0.16 ± 0.01% lactic acid, and pH 6.7 ± 0.0) was supplied from Ankara University (Ankara, Turkiye). In the study, *Lb. acidophilus*, *Lcb. rhamnosus*, and *Lcb. paracasei* (Maysa, Istanbul, Turkiye) were used as probiotic bacteria. Sodium hexametaphosphate (Sigma Aldrich, USA) was supplied in crystal form with 96% purity. Inulin (Orafti-Gr, Beneo, Mannheim, Germany) used in the study was obtained in native form.

### Production of cross-linked inulin

For chemical cross-linking of inulin, 20 g of inulin was dissolved in 300 mL of water. This solution was then stirred using a magnetic stirrer (Heidolph MR HEI TEC, Schwabach, Germany) at 500 rpm for 1 h at 25 °C. To initiate cross-linking, 0.75% SHMP was added to the inulin solution. The pH was then adjusted to 10 using 2 M Na_2_CO_3_. The mixture was waited in a shaking incubator (Mikrotest, MCI-55, Turkiye) at 45 °C for 3.5 h to allow the cross-linking reaction to proceed. Then, the reaction was terminated by adjusting the pH to 7 using 2 M HCl. The mixture was then centrifuged at 10,000 rpm for 20 min to separate the cross-linked inulin. The precipitate was subsequently washed with ethanol followed by distilled water for purification. Finally, the cross-linked inulin was lyophilized (Christ Alpha 1–2 LD, Germany) to obtain the dry product (Li et al. 2019a, b).

### Synbiotic dairy beverage production

For the production of synbiotic dairy beverages, firstly raw cow milk was pasteurized (95 °C 10 min) and either cross-linked or natural inulin were added. Then probiotic bacteria were added and incubated at 37 °C until the pH value reached 4.6. Incubation time was different for each bacteria (approximately 15, 16 and 18 h for *Lb. acidophilus*, *Lcb. rhamnosus* and *Lcb. paracasei*, respectively). The addition of cross-linked inulin did not affect the incubation time. At the end of incubation, samples were stored at 4 °C and analyzed on the 1st, 15th and 30th days. The study was carried out in 2 replicates. The experimental samples were produced following the process given in Fig. S2.

Sample codes are given below.

LA: Control sample produced with *Lb. acidophilus* and natural inulin.

LR: Control sample produced with *Lcb. rhamnosus* and natural inulin.

LC: Control sample produced with *Lcb. paracasei* and natural inulin.

LA-C: Sample produced with *Lb. acidophilus* and cross-linked inulin.

LR-C: Sample produced with *Lcb. rhamnosus* and cross-linked inulin.

LC-C: Sample produced with *Lcb. paracasei* and cross-linked inulin.

### Determination of compositional properties

Dry matter and ash content were assessed using the gravimetric method (ISO and IDF 2010). The Gerber method was employed to measure fat content (IDF 2018), while total protein was determined using the Kjeldahl method (IDF 2001). Titratable acidity was measured titrimetrically, and pH values were obtained using a digital pH-meter with combined electrodes (Ohaus, ST300, USA).

### Determination of color properties

Color analysis was performed using a colorimeter (Ser-Lab SL400, Turkiye). Measurements were taken in the CIE color space, which provides L^*^ (lightness), a^*^ (red-green), and b^*^ (yellow-blue) values.

To quantify the color differences between the cross-linked inulin samples (LA-C, LR-C, and LC-C) and their respective control samples (LA, LR, and LC), the total color difference (∆E) was calculated using formula given below. This calculation provides a numerical representation of the overall color change resulting from the use of cross-linked inulin in the synbiotic dairy beverage formulations.

$$ {\rm{\Delta E}}\,{\rm{ = }}\,\surd \left[ {{{\left( {{{\rm{L}}_{\rm{2}}}^{\rm{*}}{\rm{ - }}{{\rm{L}}_{\rm{1}}}^{\rm{*}}} \right)}^{\rm{2}}}{\rm{ + }}{{\left( {{{\rm{a}}_{\rm{2}}}^{\rm{*}}{\rm{ - }}{{\rm{a}}_{\rm{1}}}^{\rm{*}}} \right)}^{\rm{2}}}{\rm{ + }}{{\left( {{{\rm{b}}_{\rm{2}}}^{\rm{*}}{\rm{ - }}{{\rm{b}}_{\rm{1}}}^{\rm{*}}} \right)}^{\rm{2}}}} \right] $$

Where:

- L_1_*, a_1_*, b_1_* are the values of the control samples (LA, LR, or LC).
- L_2_*, a_2_*, b_2_* are the values of the corresponding cross-linked inulin samples (LA-C, LR-C, or LC-C).

### Enumeration of bacterial populations

Microbiological analyses were performed to evaluate the viability and number of both probiotic bacteria and lactobacilli and lactococci remaining in the sample after heat treatment according to Tharmaraj and Shah (2003). 10 mL of each sample diluted with Ringer’s solution. The samples were then homogenized using a laboratory mixer (Bag Mixer 400 VW, Interscience, France) for 2 min, followed by the preparation of serial dilutions. Specific bacterial enumeration methods were employed for different probiotic strains:

- *Lactobacillus* spp.: Enumerated using MRS Agar medium, incubated anaerobically at 37 °C for 72 h.
- *Lactococcus* spp.: Counted using M17 agar medium, incubated aerobically at 37 °C for 48 h.
- *Lb. acidophilus*: Determined using MRS-Sorbitol agar medium, incubated anaerobically at 37 °C for 72 h.
- *Lcb. rhamnosus*: Enumerated using MRS-Vancomycin agar medium, incubated anaerobically at 43 °C for 72 h.
- *Lcb. paracasei*: Counted using MRS-Vancomycin agar medium, incubated anaerobically at 37 °C for 72 h.

### Determination of total phenolic contents and antioxidant capacity

The total phenolic contents were determined by Folin-Ciocalteu method. The procedure involved centrifuging 10 g of each sample at 12,000 rpm for 10 min. From the centrifuged sample, 1 mL was combined with 1 mL ethyl alcohol (95%), 5 mL water, and 0.5 mL Folin-Ciocalteu solution (50%). This mixture was kept in the dark for 5 min, after which 1 mL of Na_2_CO_3_ (5%) was added to create alkaline conditions. The solution was left to react for 1 h. Following this incubation period, absorbance values were measured at 725 nm wavelength. The phenolic compound contents were then calculated as GAE using a calibration curve (Fig. S1).

Antioxidant activity of samples was determined by the DPPH (1,1-Diphenyl-2-Picrylhydrazyl) method (Apostolidis et al. 2006). Sample (10 g) was centrifuged (12000 rpm for 10 min) and filtered (Whatman No. 40). To 100 μL of the filtrate, 2 μL of 0.1 mM DPPH solution was added. The mix was incubated for 30 min, after which its absorbance was measured at 517 nm using a spectrophotometer (Lambda 25 UV/Vis, PerkinElmer, Singapore). The antioxidant activity of synbiotic beverages samples was calculated as given below.

$$ {\rm{\% }}\,{\rm{AC = }}\left( {{{\rm{A}}_{\rm{1}}}{\rm{- }}{{\rm{A}}_{\rm{2}}}} \right){\rm{/}}{{\rm{A}}_{\rm{1}}}{\rm{ \times 100}} $$

Where,

A_1_ = Absorbance of the control sample.

A_2_ = Absorbance of the test sample.

#### Determination of physical properties

Water holding capacity (WHC) of the samples was determined according to the method described by Isanga and Zhang (2009). The values were calculated as given below.

$$ {\rm{WHC}}\,\left( {\rm{\% }} \right){\rm{ = }}\left( {{\rm{1 - }}{{\rm{A}}_{\rm{c}}}{\rm{/}}{{\rm{A}}_{\rm{s}}}} \right){\rm{ \times 100}} $$

Where:

A_c_ = Weight of serum after centrifugation.

A_s_ = Initial weight of the sample.

A dynamic rheometer (Malvern Panalytical, Malvern, UK) equipped with a 40 mm, 4° angle cone and plate geometry was used for analysis. Shear stress values were measured at 28 shear rates ranging from 0.5 to 100 s^− 1^ at 5 °C. Rheological data were fitted to the Power-Law flow model given below, which yielded the highest correlation values (0.9877–0.9992):

$$ {\rm{\sigma }}\,{\rm{ = }}\,{\rm{K}} \cdot {\gamma ^{\rm{n}}} $$

Where,

σ = Shear stress.

K = Consistency index.

ɣ = Shear rate.

n = Flow behavior index.

Oscillatory analyses were performed on the samples within a frequency range of 0.01 to 10 Hz, at 5 °C, and with a shear stress of 1%. These analyses yielded values for the storage (elastic) modulus (G’) and loss (viscous) modulus (G’’).

### Determination of electrophoretic profile

The electrophoretic profile was determined using the SDS-PAGE method. Samples were diluted to a final protein concentration of 50 μg/μL and mixed with 2X sample buffer in a 1:1 ratio. After a 40-min pre-run, 15 μL of each sample was loaded onto a gel consisting of a 12.5% separating gel and a 4.5% stacking gel. Electrophoresis was conducted at 200 mV.

### Determination of sensorial properties

Sensory evaluation was performed by 7 panellists (26–47 age and 5 female-2 male) who are experienced in the sensory evaluation of dairy products, consisting of members of the Department of Dairy Technology. A 9-point hedonic scale was used (1: Strongly Disliked, 2: Strongly Disliked, 3: Moderately Disliked, 4: Slightly Disliked, 5: Neither Liked nor Disliked, 6: Slightly Liked, 7: Moderately Liked, 8: Very Much Liked, 9: Strongly Liked) according to the method described by Clark and Costello (2016).

### Statistical analysis

The experiment was performed in duplicate. Statistical analysis was performed using a randomized block design analysis of variance (ANOVA). After verifying that the data met the necessary assumptions for ANOVA, the analysis was conducted using Minitab^®^ software version 16.1.1. Tukey’s comparison test was selected to detect differences between multiple groups. *P* < 0.05 was accepted as significance level.

## Results and discussions

### Compositional properties

The research findings showed that the use of different probiotic bacteria or cross-linking of inulin did not significantly affect the compositional properties of the samples (Table 1). However, the addition of inulin led to notable changes in certain compositional aspects. Specifically, there was an observed increase in total solid and ash content. Conversely, the proportional amounts of protein and fat were found to be slightly lower than those in the raw milk. According to the results obtained in the current study, cross-linked inulin, which has technological advantages such as not changing the composition of the samples and not affecting the shelf life, can be applied for the production of functional synbiotic beverages.

**Table 1:** Compositional properties of synbiotic dairy beverages produced with natural and cross-linked inulin

Samples	Total solid (%)	Ash (%)	Protein (%)	Fat (%)
LA	15.23 ± 0.15	0.66 ± 0.02	3.19 ± 0.02	3.10 ± 0.00
LA-C	15.23 ± 0.03	0.72 ± 0.01	3.17 ± 0.02	3.15 ± 0.15
LR	15.28 ± 0.06	0.63 ± 0.02	3.19 ± 0.03	3.20 ± 0.00
LR-C	15.34 ± 0.02	0.72 ± 0.00	3.15 ± 0.08	3.16 ± 0.04
LC	15.19 ± 0.06	0.70 ± 0.06	3.19 ± 0.02	3.15 ± 0.15
LC-C	15.21 ± 0.07	0.72 ± 0.00	3.22 ± 0.01	3.15 ± 0.15

The difference was not found to be significant in the row or column without letters

### Acidity and color determination

No significant differences in acidity values were observed among synbiotic dairy beverages regardless of probiotic strain or inulin type (Table 2). Initial pH values on day 1 of storage were close to 4.6, the point at which incubation was ended during production. Although acidity did not increase significantly throughout storage, by day 30, pH values were lower and SH values higher compared to the 1st day storage.

**Table 2:** Acidity, color, total phenolic and antioxidant capacity values ​​of synbiotic dairy beverages produced with natural and cross-linked inulin

	Period of storage				
	Samples	Day 1	Day 15	Day 30	
pH value	LA	4.55 ± 0.02	4.58 ± 0.02	4.54 ± 0.02	
	LA-C	4.62 ± 0.01	4.58 ± 0.06	4.54 ± 0.05	
	LR	4.53 ± 0.13	4.43 ± 0.09	4.41 ± 0.06	
	LR-C	4.61 ± 0.02	4.58 ± 0.04	4.57 ± 0.02	
	LC	4.62 ± 0.02	4.55 ± 0.03	4.53 ± 0.08	
	LC-C	4.58 ± 0.01	4.59 ± 0.02	4.56 ± 0.02	
Titratable acidity (°SH)					
	LA	31.09 ± 0.08	31.14 ± 0.26	33.16 ± 0.60	
	LA-C	29.08 ± 1.29	31.58 ± 0.10	33.61 ± 1.11	
	LR	31.47 ± 0.56	33.35 ± 0.98	35.07 ± 0.55	
	LR-C	29.02 ± 0.09	31.56 ± 0.56	33.04 ± 0.05	
	LC	28.58 ± 0.83	31.84 ± 0.82	34.88 ± 1.13	
	LC-C	29.26 ± 0.51	31.32 ± 0.60	32.92 ± 0.60	
L*					
	LA	81.15 ± 0.35	81.20 ± 0.00	81.05 ± 0.15	
	LA-C	80.80 ± 0.20	80.90 ± 0.10	81.80 ± 0.10	
	LR	80.85 ± 0.05	81.05 ± 0.05	81.40 ± 0.20	
	LR-C	80.52 ± 0.62	81.05 ± 0.15	80.90 ± 0.50	
	LC	80.55 ± 0.65	80.75 ± 0.15	80.80 ± 0.30	
	LC-C	80.30 ± 1.00	80.80 ± 0.40	80.90 ± 0.20	
a*					
	LA	0.9 ± 0.00	1.2 ± 0.05	1.05 ± 0.05	
	LA-C	1.1 ± 0.05	1.3 ± 0.05	1.10 ± 0.00	
	LR	1.0 ± 0.00	1.2 ± 0.00	1.05 ± 0.15	
	LR-C	1.1 ± 0.05	1.3 ± 0.00	1.15 ± 0.05	
	LC	1.1 ± 0.05	1.2 ± 0.05	1.20 ± 0.00	
	LC-C	1.2 ± 0.10	1.3 ± 0.10	1.25 ± 0.05	
b*					
	LA	6.65 ± 0.05	6.60 ± 0.00	6.60 ± 0.00	
	LA-C	6.30 ± 0.00	6.25 ± 0.35	6.60 ± 0.00	
	LR	6.65 ± 0.05	6.40 ± 0.20	7.05 ± 0.35	
	LR-C	6.55 ± 0.15	6.10 ± 0.20	7.00 ± 0.00	
	LC	6.60 ± 0.30	6.60 ± 0.10	6.55 ± 0.35	
	LC-C	6.35 ± 0.25	6.25 ± 0.05	6.80 ± 0.00	
ΔE					
	LA-C	0.595 ± 0.053^B^	0.597 ± 0.042^B^	0.550 ± 0.050^B^	
	LR-C	0.912 ± 0.015^A^	0.914 ± 0.040^A^	0.939 ± 0.064^A^	
	LC-C	0.597 ± 0.065^B^	0.521 ± 0.039^B^	0.589 ± 0.025^B^	
Antioxidant capacity					
	LA	7.28 ± 0.44^BCb^	8.55 ± 0.34^Aa^	8.19 ± 0.05^Aab^	
	LA-C	7.17 ± 0.34^Bb^	8.60 ± 0.10^Aa^	7.88 ± 0.15^ABab^	
	LR	9.18 ± 0.30^Aa^	7.58 ± 0.15^ABb^	8.09 ± 0.82^Aab^	
	LR-C	9.06 ± 0.44^Aa^	7.92 ± 0.20^ABab^	5.68 ± 0.51^BCb^	
	LC	8.99 ± 0.20^ABa^	6.89 ± 0.15^BCb^	7.88 ± 0.34^ABab^	
	LC-C	8.55 ± 0.32^ABa^	6.26 ± 0.39^Cb^	4.87 ± 0.04^Cb^	
Phenolic compound (mg GAE/100 g)					
	LA	36.91 ± 3.19	36.25 ± 3.75	45.31 ± 0.59	
	LA-C	40.17 ± 1.76	38.09 ± 3.26	47.98 ± 2.31	
	LR	44.17 ± 3.85	43.18 ± 2.91	45.01 ± 2.27	
	LR-C	37.90 ± 3.40	33.98 ± 0.26	46.95 ± 1.36	
	LC	45.60 ± 2.49	40.61 ± 1.47	46.26 ± 2.05	
	LC-C	36.44 ± 1.91^b^	39.73 ± 0.07^ab^	46.73 ± 1.43^a^	

Different lowercase letters in the same row indicate differences between storage days (*P* < 0.05), and different uppercase letters in the same column indicate differences between samples (*P* < 0.05). The difference was not found to be significant in the row or column without letters

Color analysis revealed no significant differences between samples produced using different probiotic bacteria. The addition of cross-linked inulin did not substantially alter the L^*^, a^*^, and b^*^ values. Total color difference calculations confirmed this observation, with ΔE values between synbiotic dairy beverages containing cross-linked and natural inulin ranging from 0.550 to 0.939. ΔE values below 1.5 indicate minimal color difference. While samples produced by *Lcb. rhamnosus* showed slightly higher ΔE values (*p < 0.05*), these remained below 1.5, suggesting no visually perceptible change. Color values remained stable throughout 30 days of storage.

### Phenolic content and antioxidant capacity

Table 2 presents the antioxidant capacity and phenolic compound contents of the samples. On the 1st day of storage, significant differences were observed in antioxidant capacity among synbiotic dairy beverages produced with different probiotics. Beverages containing *Lb. acidophilus* showed significantly lower (*p < 0.05*) antioxidant capacity, while beverages containing *Lcb. rhamnosus* showed the highest antioxidant capacity. This findings aligns with El-Sayed et al. (2021) study, which reported *Lcb. rhamnosus* having the highest initial antioxidant activity. Interestingly, as storage progressed, beverages containing either natural or cross-linked inulin (LA and LA-C) produced with *Lb. acidophilus* showed the highest antioxidant capacity on the 15th and 30th days of storage, although no statistical difference was found. Previous studies have reported significant increases in antioxidant capacity in probiotic-containing samples after 28 days of storage, likely due to the release of bioactive fractions through bacterial proteolysis.

Conversely, the *Lcb. paracasei* sample with cross-linked inulin (LC-C) showed the lowest antioxidant capacity at the end (30th day) of storage. Cross-linked inulin containing samples with *Lcb. rhamnosus* (LR-C) and *Lcb. paracasei* (LC-C) experienced significant decreases in antioxidant capacity during storage (*p < 0.05*). This decrease may be attributed to the degradation of bioactive components during storage, as reported in previous studies on dairy products (Hala et al. 2010).

The antioxidant activity mechanisms vary among *Lactobacillus* species. *Lb. acidophilus* increases plasma folate levels (Ruiz Rodríguez et al. 2019), *Lcb. rhamnosus* inhibits NADPH oxidase enzyme and causes mRNA downregulation (Adikari et al. 2021), while *Lcb. paracasei* chelates metal ions. These varied mechanisms contribute to differences in antioxidant capacity among samples. Antioxidant properties also depend on factors such as amino acid sequence, physical structure, hydrophobicity, and molecular weight of the bacteria (Barac et al. 2019). A comparative study of seven *Lactobacillus* species reported varying antioxidant capacities, with *Lcb. paracasei* showing the lowest activity (Ramesh et al. 2012), consistent with our findings.

The cross-linking of inulin did not significantly affect antioxidant capacity on days 1 and 15 of storage. However, by the final day of storage, samples with cross-linked inulin exhibited lower antioxidant capacity compared to those with natural inulin, particularly in *Lcb. rhamnosus* and *Lcb. paracasei* samples (*p < 0.05*). Antioxidant compounds in foods often decrease during storage due to complexation with carbohydrates, proteins, or metal ions, modification by oxidative enzymes, or oxidation by available O_2_ (Starowicz and Zieliński 2019). In our study, the decreased antioxidant capacity in cross-linked inulin samples may result from antioxidant compounds forming complexes with peptides produced during storage-induced proteolysis, catalyzed by the cross-linking agent. The decrease in antioxidant capacity during storage is attributed to the increased degradation of phenolic compounds with antioxidant activity and the increased interaction of milk proteins and polyphenols (Yuksel et al. 2010). Similar to this study, there are studies on different dairy products in which the antioxidant capacity decreases during storage (Najgebauer-Lejko, 2014; Karaaslan 2011). The decrease in antioxidant capacity during storage may be due to the influence of oxygen and presence of metabolic enzyme in lactic acid bacteria (Du, 2022).

No significant differences in phenolic content were observed among the samples. On the first day of storage, samples produced with cross-linked inulin showed slightly lower phenolic content compared to those with natural inulin, except for samples containing *Lb. acidophilus* (LA and LA-C). It is thought that the reason for the lower phenolic content in samples with cross-linked inulin at the beginning of storage may be the complex formation of phenolic compounds with the cross-linking agent found in these samples, as stated in the antioxidant capacity. But, by the end of the 30-day storage period, all samples exhibited an increase in phenolic content compared to day 1. However, this increase was statistically significant only in the LC-C sample (*p < 0.05*).

The effect of inulin cross-linking on phenolic content changes during storage appears to vary depending on the bacterial culture. Initially, samples containing cross-linked inulin had lower phenolic content compared to those containing native inulin, whereas at the end of the storage period, the phenolic content levels in all samples converged, regardless of the inulin type.

### Physical properties

Water holding capacity is crucial for preventing serum separation and maintaining product stability in milk-based beverages. WHC is inversely related to syneresis, which is the release of liquid due to gel contraction in gel-structured products. Changes in WHC during storage significantly impact the quality of fermented beverages. In fermented dairy products, cold storage typically leads to a decrease in WHC and an increase in syneresis due to the disruption of the protein network structure.

This study, which investigated the effect of cross-linked inulin on synbiotic dairy beverages produced with different probiotics, observed an expected decrease in WHC during storage. The primary factor influencing WHC in fermented beverages is the acidity level of samples, with increased acidity leading to decreased WHC. As shown in Table 2, the acidity of the samples increased during storage, likely contributing to the observed decrease in WHC (Table 3). When comparing the WHC of samples with natural inulin and cross-linked inulin, the cross-linked inulin consistently increased WHC across all samples and storage days. However, both the storage-related decrease and the differences attributed to cross-linked inulin were not statistically significant (*P > 0.05*).

**Table 3:** Lactic acid bacteria counts, water holding capacity and rheological values ​​of synbiotic dairy beverages produced with natural and cross-linked inulin

	Period of storage			
	Samples	Day 1	Day 15	Day 30
*Lactococcus*				
	LA	5.78 ± 0.07^A^	5.89 ± 0.01^A^	5.07 ± 0.00^A^
	LA-C	4.97 ± 0.19^AB^	5.02 ± 0.03^B^	4.96 ± 0.02^AB^
	LR	5.04 ± 0.26^AB^	5.00 ± 0.02^B^	4.75 ± 0.03^AB^
	LR-C	4.90 ± 0.27^AB^	4.30 ± 0.12^B^	4.58 ± 0.16^BC^
	LC	4.40 ± 0.22^B^	4.61 ± 0.10^B^	4.30 ± 0.07^CD^
	LC-C	4.77 ± 0.31^AB^	4.43 ± 0.31^B^	4.07 ± 0.05^D^
*Lactobacillus*				
	LA	8.08 ± 0.04	7.96 ± 0.02	7.99 ± 0.08
	LA-C	7.96 ± 0.05	7.91 ± 0.05	7.91 ± 0.06
	LR	8.05 ± 0.09	7.95 ± 0.04	7.95 ± 0.08
	LR-C	7.76 ± 0.19	7.75 ± 0.07	7.78 ± 0.08
	LC	7.81 ± 0.33	7.78 ± 0.40	7.81 ± 0.38
	LC-C	7.61 ± 0.33	7.52 ± 0.10	7.55 ± 0.43
*Lb. acidophilus*				
	LA	7.98 ± 0.01^a^	7.82 ± 0.12^a^	7.51 ± 0.09^a^
	LA-C	8.00 ± 0.03^a^	7.73 ± 0.05^b^	7.73 ± 0.01^b^
*Lcb. rhamnosus*				
	LR	8.08 ± 0.05^a^	7.98 ± 0.00^ab^	7.88 ± 0.01^b^
	LR-C	7.79 ± 0.07^a^	7.74 ± 0.11^a^	7.68 ± 0.05^a^
*Lcb. paracasei*				
	LC	7.77 ± 0.07^a^	7.68 ± 0.10^a^	7.71 ± 0.15^a^
	LC-C	7.55 ± 0.05^a^	7.41 ± 0.01^b^	7.31 ± 0.08^b^
WHC (%)				
	LA	23.98 ± 0.99	23.32 ± 1.31	22.52 ± 0.36
	LA-C	25.63 ± 0.26	24.41 ± 0.10	22.84 ± 0.83
	LR	25.45 ± 0.94	25.09 ± 0.56	23.21 ± 0.95
	LR-C	25.87 ± 0.25	25.51 ± 0.26	24.03 ± 0.64
	LC	25.82 ± 0.23	22.92 ± 0.19	22.98 ± 0.08
	LC-C	26.64 ± 1.10	24.64 ± 1.16	22.06 ± 0.48
K (Pa.s)				
	LA	3.37 ± 0.20^AB^	3.46 ± 0.23^AB^	3.99 ± 0.42^B^
	LA-C	4.13 ± 0.51^A^	4.68 ± 0.41^A^	5.64 ± 0.06^A^
	LR	2.08 ± 0.09^C^	2.77 ± 0.28^B^	3.16 ± 0.51^BC^
	LR-C	2.70 ± 0.09^B^	3.55 ± 0.16^AB^	3.79 ± 0.19^BC^
	LC	2.19 ± 0.10^C^	2.32 ± 0.14^C^	2.51 ± 0.03^C^
	LC-C	2.84 ± 0.03^B^	2.92 ± 0.29^B^	2.94 ± 0.10^BC^
*n*				
	LA	0.34 ± 0.03^a^	0.29 ± 0.01^b^	0.26 ± 0.01^b^
	LA-C	0.32 ± 0.00^a^	0.25 ± 0.02^b^	0.25 ± 0.00^b^
	LR	0.55 ± 0.02^a^	0.32 ± 0.00^c^	0.30 ± 0.09^b^
	LR-C	0.39 ± 0.07^a^	0.34 ± 0.03^a^	0.34 ± 0.05^a^
	LC	0.41 ± 0.07^a^	0.37 ± 0.03^a^	0.30 ± 0.07^b^
	LC-C	0.38 ± 0.03^b^	0.30 ± 0.02^a^	0.29 ± 0.04^a^

Different lowercase letters in the same row indicate differences between storage days (*P* < 0.05), and different uppercase letters in the same column indicate differences between samples (*P* < 0.05). The difference was not found to be significant in the row or column without letters

The primary objective of incorporating cross-linked and natural inulin in this research was to enhance the physical and, particularly, the rheological properties of beverages containing different probiotic bacteria. The consistency coefficient (K) results presented in Table 3 demonstrate that cross-linked inulin positively influences the consistency properties of the samples, increasing their viscosity. However, the impact of the cross-linking process varied among sample groups. The effect was most pronounced in LA-coded samples throughout storage. Notably, the increase in K values for LA samples with cross-linked inulin became significantly more pronounced by the end of the storage period (day 30). In contrast, the effect of natural versus cross-linked inulin on K values in other samples remained consistent from the first day of storage.

Consistency coefficient values differed significantly among the sample groups produced using three different lactic acid bacteria (*p < 0.05*). Samples with *Lb. acidophilus* exhibited the highest K values. The elevated viscosity observed in these samples throughout storage is likely attributed to their potential exopolysaccharide (EPS) production. All three lactic acid bacteria used in this study are known EPS producers which has a enhancing effect on the consistency properties of foods. However, EPS production potential varies among species, and environmental conditions significantly influence EPS production levels (Nguyen et al. 2020). Environmental factors affecting EPS production include dehydration, extreme temperature, acid, osmotic stress, phagocytosis, macrophages, and antibiotics, as well as nutrients found in the environment. Depending on the species and sometimes strains of lactic acid bacteria, the type of monosaccharide found for EPS production may be important. Indeed, the study conducted by Hussein et al. (2015) reported that *Lcb. paracasei* produces more EPS in glucose-rich environments compared to lactose-rich ones. In our study, while Lcb. paracasei typically has a high EPS production potential, it unexpectedly showed the lowest thickening effect in our samples. So that, the fact to be lower in our study may be due to *Lcb. paracasei*’s preference for high-glucose environments for optimal EPS production.

The consistency coefficient values increase slightly during storage, a change generally associated with decreasing pH. Table 2 reveals a slight, though not statistically significant, decrease in pH values over time. This pH reduction promotes interaction between denatured serum proteins and paracasein. Consequently, paracasein micelle volume expands, leading to increased product viscosity (Pushpadass et al. 2019).

All samples exhibited flow behavior index (n) values below 1 (ranging from 0.25 to 0.55), indicating that neither the use of different probiotics nor cross-linked inulin altered the flow behavior. The flow remained pseudoplastic across all samples. Statistical analysis also revealed that the difference between samples was insignificant. Furthermore, a significant decrease in the *n* value corresponded with an increase in the K value during storage (*P < 0.05*).

**Fig. 1:**
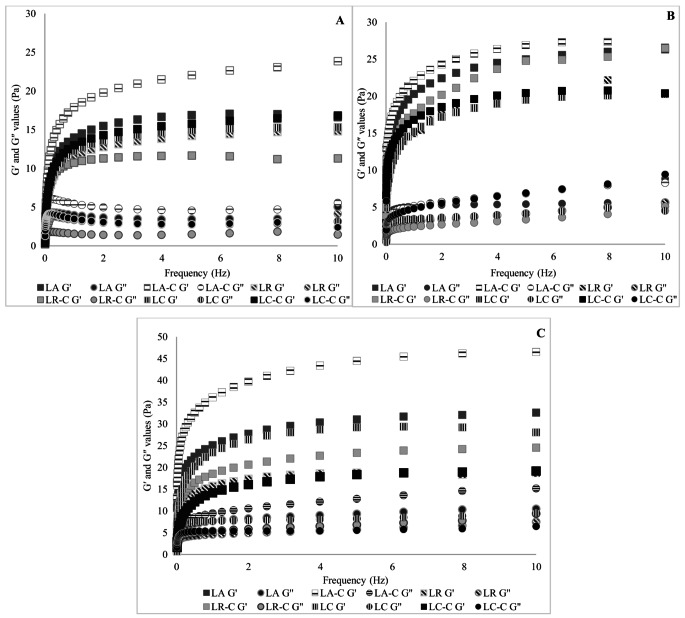
Storage (G’) and loss (G’’) modulus values ​​of synbiotic dairy beverages produced with natural and cross-linked inulin on the 1st (**A**), 15th (**B**) and 30th (**C**) storage days

Figure 1 presents the storage modulus (G′) and loss modulus (G″) values obtained during 30 days of storage, illustrating the viscoelastic properties of synbiotic dairy beverages. G′ represents the energy stored and recovered per cycle, characterizing the solid-like behavior and structural integrity of the sample. G″ measures the energy dissipated or lost per deformation cycle, indicating the liquid-like response of viscous components (Barnes 2000). Throughout the storage period, the G′ values of all synbiotic dairy beverage samples exceeded their G″ values, indicating a predominantly solid behaviour. Both modulus values increased during storage with samples reaching their peak G′ and G″ values on the 30th day of storage. The higher values ​​observed on day 30 compared to day 1 indicate that the consistency of the sample has increased. This finding aligns with the rise in K values over the 30-day storage period, as shown in Table 3. Present findings have shown that the addition of cross-linked inulin may provide stability to the product during storage without the need for additional stabilizers. In addition, the resulting increase in consistency may improve the mouthfeel of the product. This may support industrial-scale production of the product.

### Enumeration of bacterial populations

All bacteria exhibited a decline in numbers during 30 days storage (Table 3). This reduction in microorganism count in fermented products can be attributed to increasing acidity, depletion of essential nutrients, or changes in dissolved oxygen levels. Despite this decline, the specific counts of *Lb. acidophilus*, *Lcb. rhamnosus*, and *Lcb. paracasei* remained above the threshold (> 10^6^) required for a product to be considered probiotic throughout the storage period. This maintenance of probiotic levels ensures functional benefits of samples are preserved during its shelf life.

Synbiotic dairy beverage samples contained relatively low numbers of *Lactococcus* spp. The LA-coded samples exhibited the highest *Lactococcus* count, while LC-coded samples showed the lowest. This variation may be attributed to the different fermentation patterns of the probiotic strains. *Lb. acidophilus* is known to be obligately homofermentative, whereas *Lcb. rhamnosus* and *Lcb. paracasei* are facultatively heterofermentative (Dempsey and Corr 2022) observed differences in *Lactococcus* counts are likely to be due to metabolites produced by *Lcb. rhamnosus* and *Lcb. paracasei* species rather than lactic acid. These additional metabolites may influence the growth environment, potentially affecting *Lactococcus* populations in the samples.

### Electrophoretic profile

**Fig. 2:**
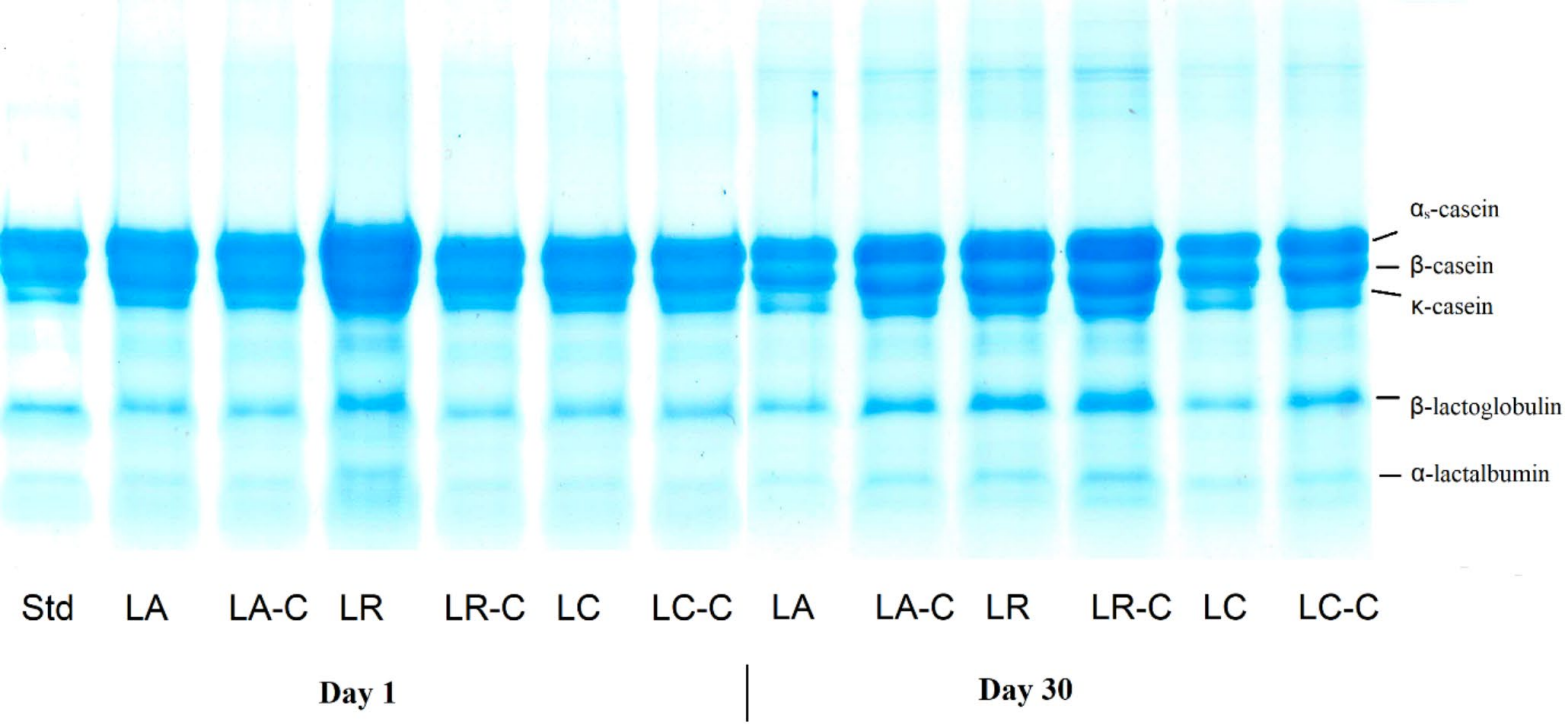
SDS-PAGE electrophoretogram of synbiotic dairy beverage samples produced with natural and cross-linked inulin on the 1st and 30th storage days

Figure 2 displays the SDS-PAGE electrophoretograms of the samples on the 1st and 30th storage days. Analysis of the 1st day results reveals that the use of cross-linked inulin and native inulin did not significantly alter the protein fractions across samples. However, the LR-coded sample exhibited a notably denser paracasein band compared to other samples. In SDS-PAGE electrophoretograms, polypeptides with hydrophobic side chain sequences typically form wider bands with indistinct edges. This study’s results suggest that samples containing *Lcb. rhamnosus* had a higher concentration of polypeptides with hydrophobic side chains. Hydrophobicity plays a crucial role in the biological properties of food-derived peptides. Previous research has linked hydrophobic peptides to high antioxidant capacity (Yao et al. 2018). Correspondingly, samples with *Lcb. rhamnosus* addition showed higher antioxidant capacity (Table 2), corroborating this relationship. By the 30th day of storage, this difference had diminished. Notably, samples with cross-linked inulin maintained band patterns similar to day 1, with the LR-C sample showing even more intense band. In contrast, samples with native inulin exhibited thinning of paracasein bands, indicating greater protein degradation compared to cross-linked inulin samples. While cross-linking of inulin did not affect acidity or microorganism counts, it influenced the extent of protein degradation. Among samples with native inulin, those using *Lb. acidophilus* as the culture showed the most pronounced thinning of the paracasein band. This observation aligns with previous studies investigating proteolytic activities of various *Lactobacillus* species, which have reported higher proteolytic activity in *Lb. acidophilus* compared to other species used in this study (Bergamini et al. 2009).

### Sensory evaluation

**Fig. 3:**
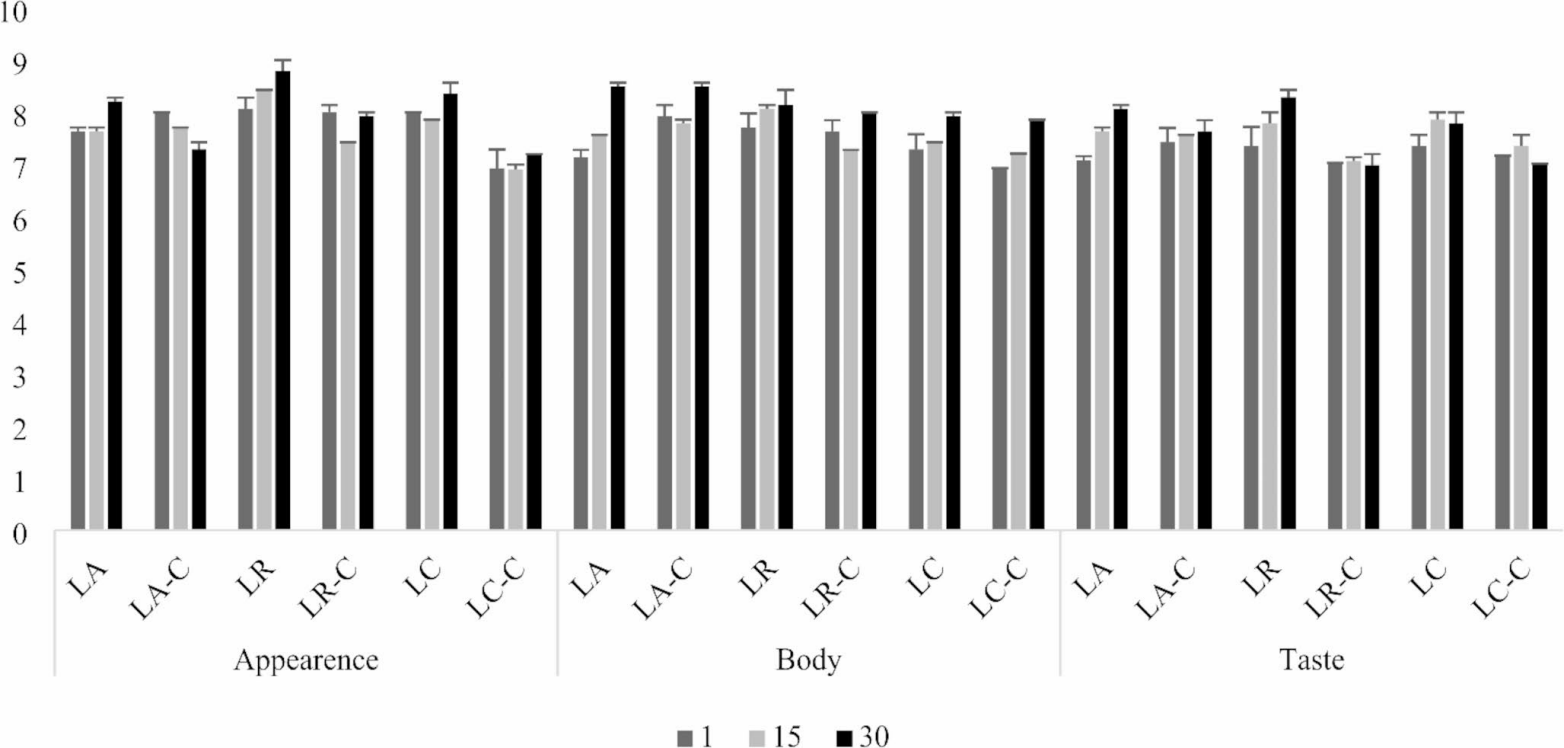
Sensory evaluation results of synbiotic dairy beverage samples produced with natural and cross-linked inulin

Sensory evaluation results (Fig. 3) indicate no significant differences among samples in terms of sensorial structure and flavor. Despite the substantial impact of inulin cross-linking on rheological properties, panelists did not perceive these changes as significant. Samples produced containing native inulin (LA, LR, and LC) showed an increase in all sensory parameter values during storage. Conversely, samples containing cross-linked inulin exhibited a slight decrease in sensory scores over storage time. However, this decrease was not statistically significant. No significant change was detected in taste in any of the samples with cross-linked inulin added during the storage. This situation reveals that the taste of the product remained stable throughout storage. In the rheological measurements, it was determined that the viscosity of all samples increased towards the end of storage (Fig. 1). The increases observed in terms of body-texture values ​​in the sensory evaluation can be attributed to this viscosity increase. Samples with *Lcb. rhamnosus* received the highest flavor scores. The addition of cross-linked inulin resulted in lower sensory scores for samples using *Lcb. paracasei* and *Lcb. rhamnosus* cultures. However, cross-linked inulin enhanced sensory scores in samples produced with *Lb. acidophilus*.

Considering all sensory evaluation parameters, all samples were found to be acceptable according to the sensory evaluation scale. This suggests that while there were subtle differences in sensory attributes, all formulations maintained satisfactory overall quality throughout the storage period.

## Conclusion

This study investigated the effects of natural and cross-linked inulin addition on synbiotic dairy beverages produced using three probiotic lactic acid bacteria (*Lb. acidophilus*, *Lcb. rhamnosus*, and *Lcb. paracasei*). The research findings revealed that different prebiotic and probiotic combinations did not significantly alter the beverages’ composition, acidity, or color values. Antioxidant capacity varied among samples with different probiotics on the first day of storage. However, as storage progressed to day 30, samples containing cross-linked inulin showed decreased antioxidant capacity. The primary goal of adding cross-linked inulin was to enhance the rheological properties of samples. As anticipated, samples with cross-linked inulin consistently exhibited higher consistency coefficient values throughout the storage period. Despite concerns that the increased molecular weight of cross-linked inulin might reduce its prebiotic effect, probiotic bacteria counts remained stable in these samples. All samples experienced a decrease in probiotic bacteria counts during the 30-day storage period. However, counts remained above the minimum threshold (> 10^6^) required for probiotic products. SDS-PAGE results indicated no significant differences in protein fractions among samples. Particularly, less proteolysis was observed in samples containing cross-linked inulin than in those containing native inulin over a 30-day storage period. Sensory evaluation demonstrated that synbiotic beverages with cross-linked inulin maintained satisfactory appearance, structure, and taste, without negative effects.

In conclusion, the use of cross-linked inulin in synbiotic dairy beverage production did not significantly impact the compositional properties but improved its rheological properties and increased viscosity. Among the probiotic bacteria investigated, cross-linking was more effective in samples produced with *Lb. acidophilus*. The study underscores the potential of cross-linked inulin to improve the textural properties and stability of synbiotic beverages, making it a promising ingredient for the functional food industry.

In future studies, investigating the mechanism of the stabilization effect of cross-linked inulin in beverages will be important in terms of optimizing the improvement effect demonstrated in this study. In addition, the effects of different cross-linked prebiotics on the physical properties of dairy beverages can be investigated and production conditions can be optimized to create a stabilizing effect.

## Electronic supplementary material

Below is the link to the electronic supplementary material.

Supplementary Material 1

## Data Availability

All experimental data and detailed experimental procedures are available upon request from the corresponding author.

## Abbreviations

*Lb.*: *Lactobacillus*
*Lcb.*: *Lacticaseibacillus*
SHMP: Sodium hexametaphosphate
DPPH: 2,2-diphenyl-1-picrylhydrazyl; di(phenyl)-(2,4,6-trinitrophenyl) iminoazanium)
WHC: Water holding capacity
G’: Storage (elastic) modulus
G’’: Loss (viscous) modulus
SDS-PAGE: Sodium dodecyl sulfate polyacrylamide gel electrophoresis

## References

[CR1] Adikari AMMU, Priyashantha H, Disanayaka JNK, Jayatileka DV, Kodithuwakku SP, Jayatilake JAMS, Vidanarachchi JK (2021) Isolation, identification and characterization of Lactobacillus species diversity from Meekiri: traditional fermented buffalo milk gels in Sri Lanka. Heliyon 7(10):08136 10.1016/j.heliyon.2021.e08136 PMC8503854 34660933

[CR2] Akal C (2023) Rheological properties of cross-linked inulin solutions as a function of cross-linker concentration and temperature. J Dispers Sci Technol 44(12):2219–2230

[CR3] Ali MS, Hussein RM, Gaber Y, Hammam OA, Kandeil MA (2019) Modulation of JNK-1/ β-catenin signaling by *Lactobacillus casei*, inulin and their combination in 1,2-dimethylhydrazine-induced colon cancer in mice. RSC Adv 9(50):29368–29383 35528422 10.1039/c9ra04388h PMC9071812

[CR4] Apostolidis E, Kwon Y-I, Shetty K (2006) Potential of select yogurts for diabetes and hypertension management. J Food Biochem 30(6):699–717

[CR5] Arruda HS, Silva EK, Pereira GA, Meireles MAA, Pastore GM (2020) Inulin thermal stability in prebiotic carbohydrate-enriched araticum whey beverage. LWT Food Sci Technol 128:109418

[CR6] Baker MT, Lu P, Parrella JA, Leggette HR (2022) Consumer acceptance toward functional foods: a scoping review. Int J Environ Res Public Health 19(3) 10.3390/ijerph19031217 PMC8835010 35162240

[CR7] Barac M, Vucic T, Zilic S, Pesic M, Sokovic M, Petrovic J, Kostic A, Sredovic Ignjatovic I, Milincic D (2019) The effect of in vitro digestion on antioxidant, ACE-Inhibitory and antimicrobial potentials of traditional Serbian White-Brined cheeses. Foods 8(3):94 30871005 10.3390/foods8030094 PMC6462927

[CR8] Barnes HA (2000) A handbook of elementary rheology. University of Wales, Institute of Non-Newtonian Fluid Mechanics

[CR9] Bergamini CV, Hynes ER, Palma SB, Sabbag NG, Zalazar CA (2009) Proteolytic activity of three probiotic strains in semi-hard cheese as single and mixed cultures: *Lactobacillus acidophilus*, *Lactobacillus paracasei* and *Bifidobacterium lactis*. Int Dairy J 19(8):467–475

[CR10] Clark S, Costello M (2016) Dairy products evaluation competitions. In: Clark S, Costello M, Drake MA, Bodyfelt F (eds) The sensory evaluation of dairy products. Springer Science Business Media, USA, pp 43–729

[CR11] da Silveira EO, Neto JHL, da Silva LA, Raposo AES, Magnani M, Cardarelli HR (2015) The effects of inulin combined with oligofructose and goat cheese whey on the physicochemical properties and sensory acceptance of a probiotic chocolate goat dairy beverage. LWT-Food Sci Technol 62(1):445–451

[CR12] Dempsey E, Corr SC (2022) Lactobacillus spp. for gastrointestinal health: current and future perspectives. Front Immunol 13:840245 35464397 10.3389/fimmu.2022.840245 PMC9019120

[CR13] Drabinska N, Jarocka-Cyrta E, Zlotkowska D, Abramowicz P, Krupa-Kozak U (2019) Daily oligofructose-enriched inulin intake impacts bone turnover markers but not the cytokine profile in pediatric patients with celiac disease on a gluten-free diet: results of a randomised, placebo-controlled pilot study. Bone 122:184–192 30840918 10.1016/j.bone.2019.03.001

[CR14] Du H, Wang X, Yang H, Zhu F, Tang D, Cheng J, Liu X (2022) Changes of phenolic profile and antioxidant activity during cold storage of functional flavored yogurt supplemented with mulberry pomace. Food Control 132:108554

[CR15] El-Sayed MI, Awad S, Abou-Soliman NHI (2021) Improving the antioxidant properties of Fermented Camel milk using some strains of *Lactobacillus*. Food Nutr Sci 12(4):20

[CR16] Fornelli AR, Bandiera NS, Costa MD, de Souza CHB, de Santana EHW, Sivieri K, Aragon-Alegro LC (2015) Effect of inulin and oligofructose on the physicochemical, microbiological and sensory characteristics of symbiotic dairy beverages. Semina: Ciências Agrárias 35(6):3099–3111

[CR17] Guimarães JD, Silva EK, Costa AL, Cunha RL, Freitas MQ, Meireles MA, Cruz AG (2018) Manufacturing a prebiotic whey beverage exploring the influence of degree of inulin polymerization. Food Hydrocolloids 77:787–795

[CR18] Hala M, El D, Ghita I, Sanaa M, Badran S, Gad A, Marwa M, El-Said M (2010) Manufacture of low fat UF-soft cheese supplemented with Rosemary extract (as natural antioxidant). J Am Sci 6

[CR19] Hussein M-DM, Ghaly MF, Osman MY, Shalaby ASG, Helal MMI (2015) Production and prebiotic activity of exopolysaccharides derived from some probiotics. Egypt Pharm J 14(1):1–9

[CR20] IDF (2001) Milk determination of nitrogen content, part 5: determination of protein-nitrogen content. In, vol standard 20–5. International Dairy Federation, Brussels

[CR21] IDF (2018) Milk-determination of fat content-acido-butyrometric (Gerber method). Standard 238. International Dairy Federation, Brussels

[CR22] Isanga J, Zhang G (2009) Production and evaluation of some physicochemical parameters of peanut milk yoghurt. LWT - Food Sci Technol 42(6):1132–1138

[CR23] ISO & IDF (2010) Milk, cream and evaporated milk - determination of total solids content (Reference method). In, vol ISO 6731:2010 IDF 21:2010. Switzerland

[CR24] Karaaslan M, Ozden M, Vardin H, Turkoglu H (2011) Phenolic fortification of yogurt using grape and callus extracts. LWT– Food Sci Technol 44:1065–1072

[CR25] Li S, Hu T, Chen Y, Zheng C, Liu T, Ma G, Su Z (2010) Cross-linked inulin as a potential plasma expander: biochemical properties and physiological characterization in a rabbit model. Carbohydr Polym 82(4):1054–1060

[CR26] Li Y, Ma X, Liu X (2019a) Physicochemical and rheological properties of cross-linked inulin with different degree of polymerization. Food Hydrocolloids 95:318–325

[CR27] Li Y, Shabani KI, Qin X, Yang R, Jin X, Ma X, Liu X (2019b) Effects of cross-linked inulin with different polymerisation degrees on physicochemical and sensory properties of set-style yoghurt. Int Dairy J 94:46–52

[CR28] Mitchell CM, Davy BM, Halliday TM, Hulver MW, Neilson AP, Ponder MA, Davy KP (2015) The effect of prebiotic supplementation with inulin on cardiometabolic health: rationale, design, and methods of a controlled feeding efficacy trial in adults at risk of type 2 diabetes. Contemp Clin Trials 45:328–337 26520413 10.1016/j.cct.2015.10.012 PMC4743874

[CR29] Montanuci FD, Pimentel TC, Garcia S, Prudencio SH (2012) Effect of starter culture and inulin addition on microbial viability, texture, and chemical characteristics of whole or skim milk kefir. Food Sci Technol 32:580–865

[CR30] Najgebauer-Lejko D, Grega T, Tabaszewska M (2014) Yoghurts with addition of selected vegetables: acidity, antioxidant properties and sensory quality. Acta Sci Pol Technol Aliment 13(1):35–42. 10.17306/j.afs.2014.1.3 24583382 10.17306/j.afs.2014.1.3

[CR31] Nguyen PT, Nguyen TT, Bui DC, Hong PT, Hoang QK, Nguyen HT (2020) Exopolysaccharide production by lactic acid bacteria: the manipulation of environmental stresses for industrial applications. AIMS Microbiol 6(4):451–469 33364538 10.3934/microbiol.2020027 PMC7755584

[CR32] Pushpadass HA, Emerald FME, Balasubramanyam BV, Patel SS (2019) 11 - Rheological Properties of Milk-based beverages. In: Grumezescu AM, Holban AM (eds) Milk-based beverages. Woodhead Publishing, pp 373–396

[CR33] Ramesh V, Kumar R, Singh RRB, Kaushik JK, Mann B (2012) Comparative evaluation of selected strains of lactobacilli for the development of antioxidant activity in milk. Dairy Sci Technol 92(2):179–188

[CR34] Ruiz Rodríguez LG, Mohamed F, Bleckwedel J, Medina R, De Vuyst L, Hebert EM, Mozzi F (2019) Diversity and functional properties of lactic acid Bacteria isolated from wild fruits and flowers Present in Northern Argentina. Front Microbiol 10 10.3389/fmicb.2019.01091 PMC6536596 31164879

[CR35] Starowicz M, Zieliński H (2019) Changes in the antioxidant capacity and polyphenols content of rye- buckwheat cakes fortified with spices during their long-term storage. Ital J Food Sci 31:253

[CR36] Tharmaraj N, Shah NP (2003) Selective Enumeration of *Lactobacillus delbrueckii* ssp. *bulgaricus*, *Streptococcus thermophilus*, *Lactobacillus acidophilus*, *Bifidobacteria*, *Lactobacillus casei*, *Lactobacillus rhamnosus*, and Propionibacteria. Journal of Dairy Science. 86(7), 2288–2296 10.3168/jds.S0022-0302(03)73821-1 12906045

[CR37] Yao S, Agyei D, Udenigwe CC (2018) Chapter four - structural basis of Bioactivity of Food Peptides in promoting Metabolic Health. In: Toldrá F (ed) Advances in Food and Nutrition Research, vol 84. Academic, pp 145–181 10.1016/bs.afnr.2017.12.002 29555068

[CR38] Yuksel Z, Avci E, Erdem YK (2010) Characterization of binding interactions between green tea flavanoids and milk proteins. Food Chem 121:450–456. 10.1016/j.foodchem.2009.12.064

