# Supplementary material for: Enhancing synbiotic dairy beverages with chemically cross-linked inulin for improved texture and stability
Source: J Food Sci Technol. 2025 Mar 7;63(4):740–52. doi: 10.1007/s13197-025-06243-w (PMC13013743; doi:10.1007/s13197-025-06243-w)
Supplement: Supplementary file 1 — Supplementary Material 1 [file 13197_2025_6243_MOESM1_ESM.docx]

Raw Cow Milk

Pasteurisation (95°C 10 min)

Cooling (50°C)

Inulin Addition (4% (w/v)- Natural or cross-linked form)

Cooling (37°C)

Inoculation

(strain *Lb. acidophilus* (LA), *Lb. rhamnosus* (LR) or *Lb. casei* (LC))

Incubation (37°C-pH 4.6)

Cooling (4°C)

Storage (4°C)

**Fig. S1**. Synbiotic dairy beverage produced with natural and cross-linked inulin production

**Fig. S2.** Calibration curve of gallic acid standard
